# An Innovative, Paradigm-Shifting Lifestyle Intervention to Reduce Glucose Excursions With the Use of Continuous Glucose Monitoring to Educate, Motivate, and Activate Adults With Newly Diagnosed Type 2 Diabetes: Pilot Feasibility Study

**DOI:** 10.2196/34465

**Published:** 2022-02-23

**Authors:** Tamara K Oser, Mark Cucuzzella, Marilyn Stasinopoulos, Matthew Moncrief, Anthony McCall, Daniel J Cox

**Affiliations:** 1 Department of Family Medicine University of Colorado School of Medicine Aurora, CO United States; 2 Department of Family Medicine West Virginia University School of Medicine Morgantown, WV United States; 3 Department of Psychiatry and Neurobehavioral Sciences University of Virginia School of Medicine Charlottesville, VA United States; 4 Department of Medicine: Endocrinology and Metabolism University of Virginia School of Medicine Charlottesville, VA United States

**Keywords:** type 2 diabetes, continuous glucose monitoring, glycemic excursion minimization, initial treatment, diabetes distress, diabetes, monitoring, treatment, distress, pilot study, lifestyle, intervention, motivation.

## Abstract

**Background:**

Type 2 diabetes (T2D) is a growing epidemic in the United States, and metabolic control has not been improved over the last 10 years. Glycemic excursion minimization (GEM) is an alternative lifestyle treatment option focused on reducing postnutrient glucose excursions rather than reducing weight. GEM has been proven to be superior to routine care when delivered face to face, and equivalent or superior to conventional weight loss therapy, but it has not been evaluated among patients newly diagnosed with T2D or in a self-administered format.

**Objective:**

This pilot study evaluated the feasibility of a self-administered version of GEM, augmented with continuous glucose monitoring (CGM), to improve metabolic control (hemoglobin A_1c_ [HbA_1c_]) while diminishing or delaying the need for diabetes medications in adults recently diagnosed with T2D. These primary objectives were hypothesized to be achieved by reducing carbohydrate intake and increasing physical activity to diminish CGM glucose excursions, leading to the secondary benefits of an increase in diabetes empowerment and reduced diabetes distress, depressive symptoms, and BMI.

**Methods:**

GEM was self-administered by 17 adults recently diagnosed with T2D (mean age 52 years, SD 11.6 years; mean T2D duration 3.9 months, SD 2.5 months; mean HbA_1c_ levels 8.0%, SD 1.6%; 40% female; 33.3% non-White), with the aid of a 4-chapter pocket guide and diary, automated motivational text messaging, and feedback from an activity monitor, along with CGM and supplies for the 6-week intervention and the 3-month follow-up. Treatment was initiated with one telephone call reviewing the use of the technology and 3 days later with a second call reviewing the use of the GEM pocket guide and intervention.

**Results:**

At 3-month follow-up, 67% of the participants’ diabetes was in remission (HbA_1c_ levels <6.5%), and only one participant started taking diabetes medication. Participants demonstrated a significant reduction in HbA_1c_ levels (–1.8%; *P*<.001). Participants also experienced significant reductions in high-glycemic-load carbohydrates routinely consumed, CGM readings that were >140 mg/dL, diabetes distress, depressive symptoms, and BMI. Participants felt that use of the CGM was the most significant single element of the intervention.

**Conclusions:**

GEM augmented with CGM feedback may be an effective initial intervention for adults newly diagnosed with T2D. A self-administered version of GEM may provide primary care physicians and patients with a new tool to help people recently diagnosed with T2D achieve remission independent of medication and without weight loss as the primary focus. Future research is needed with a larger and more diverse sample.

## Introduction

Type 2 diabetes (T2D) is an epidemic in the United States, with 1.5 million new cases diagnosed annually, 34 million patients currently with the condition, and costing the US economy US $327 billion annually [1]. While the American Diabetes Association recommends weight loss of at least 7% and at least 150 minutes per week of moderate to vigorous physical activity to help manage T2D [2], up to 15% of these patients do not need to lose weight [3], while others are either unwilling or unable to lose weight and to maintain weight loss [4]. Further, glycemic control has not improved over the past decade [5-7]. International and national guidelines recommend diabetes self-management education and support (DSMES) for people with T2D around the time of diagnosis, but DSMES at the time of diagnosis is severely underutilized (only 6.8% of privately insured individuals and 5% of Medicare individuals receive DSMES within the first 12 months of being diagnosed with T2D) [8]. We developed an alternative lifestyle treatment option, glycemic excursion minimization (GEM), focusing on reducing postnutrient blood glucose (BG) excursions that contribute to both hemoglobin A_1c_ (HbA_1c_) [9] and to cardiovascular disease [10,11]. Although not classified as DSMES, GEM presents an additional method that may empower people with diabetes to better understand the impact of food and exercise on their blood glucose levels. GEM has been administered as a face-to-face intervention to adults diagnosed with T2D within the past 10 years and was superior to routine care [12,13] and equivalent or superior to conventional weight loss therapy in regard to reduction of HbA_1c_ levels, cardiovascular risk, and improvement in psychological function and BMI [14,15]. However, GEM has never been evaluated among patients newly diagnosed with T2D, in a self-administered format, outside of the University of Virginia, with automated daily text prompts. Such an intervention might not only improve metabolic control but also reduce the reliance on diabetes medications.

Given accessibility issues owing to the pandemic, we investigated the efficacy of a remote GEM delivery program. Given that earlier intervention of T2D has greater long-term benefits [16] and may provide access to more motivated persons [17], we focused on newly diagnosed patients. Given that GEM has only been evaluated at the University of Virginia, we delivered GEM at diverse medical settings (external validity). Given that daily text messages have been demonstrated to improve engagement by adults with T2D [18], we employed text messaging for the first time. The primary hypotheses tested were that GEM combined with feedback from continuous glucose monitors (CGMs) and activity monitors, with automated text messages could improve metabolic control with reduced reliance on diabetes medication, while producing the secondary benefits of improved psychological function and reduced BMI.

## Methods

### Ethics Approval

This study was performed in accordance with the principles of the Declaration of Helsinki. Ethical approval for this study was obtained from the University of Virginia Institutional Review Board for Health Sciences Research (protocol HSR200250).

### Recruitment

To maximize external validity, one-third of the participants were recruited from each of three diverse centers: University of Virginia (n=5), University of Colorado (n=6), and West Virginia University (n=6). Inclusion criteria were as follows: age of 35-85 years, HbA_1c_ levels of 6.5%-11.5%, diagnosed with T2D within the past 12 months, not taking diabetes medication, no medical condition or medication that precludes reducing carbohydrates or walking 120 steps per minute for 10 minutes (eg, prednisone, severe neuropathy, cardiovascular disease, chronic obstructive pulmonary disease or emphysema, osteoarthritis, stroke, severe gastroparesis, ulcers, or food allergies), and ablilty to read English. Our age criteria aimed to select individuals most likely to be in control of their daily routine, HbA_1c_ criteria aimed to ensure the diagnoses of T2D but to avoid individuals whose condition was so progressive that immediate medication management was indicated, the no diabetes medication criterion was essential to test the hypothesis that GEM would prevent or diminish the need for diabetes medication, and the remaining criteria aimed to ensure the feasibility of engaging in the comprehensive GEM lifestyle.

### Procedure

After signing a University of Virginia IRB-approved consent, each participant’s primary care physician or clinician was contacted to affirm that the participant met eligibility criteria and to provide written approval for participation. Next, participants were sent a weblink to complete a series of questionnaires (Baseline: routine consumption of high and low glycemic load foods [19]; psychological questionnaires to assess diabetes empowerment [20], diabetes distress (emotional and regimen) [21], and depressive symptoms [22]; and diabetes knowledge as it relates to GEM principles [23].

After completing questionnaires, participants were mailed a CGM reader and 4.5 months of sensor supplies, (FreeStyle Libre 2 CGM system), a Fitbit Charge 3 activity monitor, and the GEM pocket guide (hard copy). This was followed by a telephone call introducing them to the CGM and activity monitor technology, inserting the CGM sensor, registering and activating the technology, and selecting seven personalized text messages, for example, “Food choices are life choices, Exercise is my friend,” which would be delivered at a time and frequency selected by the participant, to encourage GEM engagement [18]. Three days later, they received the second and final call to review the GEM pocket guide to initiate treatment.

**Figure 1:**
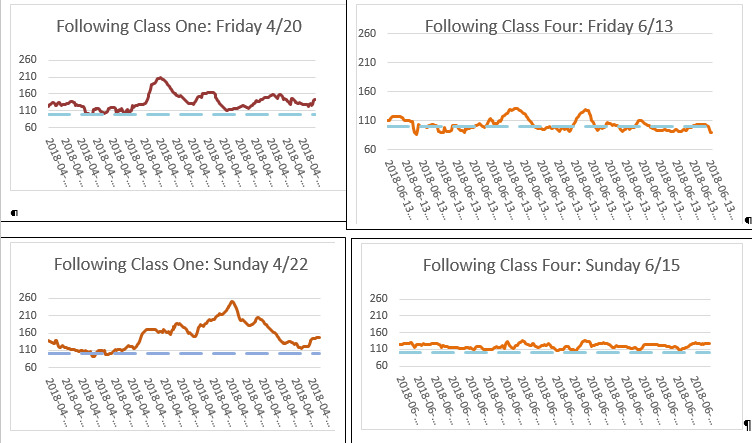
Continuous glucose monitoring data from one participant at the beginning and end of glycemic excursion minimization: change in the area under the receiver operating characteristic curve 27,600 to 8475 and change in hemoglobin A_1c_ levels 8.8% to 5.7%.

GEM is neither a behavior modification nor a prescription program. Rather, GEM is an empowerment program [24,25] that provides information that a patient can choose to employ, to identify food and activity choices that either exacerbate or diminish postnutrient glucose excursions (represented by the area under the curve in Figure 1). The GEM pocket guide is a 4.25×5.50-inch booklet with four units. In unit 1, with CGM feedback, participants spend 5 days learning which of their routine food and physical activity choices have major and minor impacts on their glucose excursions. In unit 2 participants spend 14 days focusing on reducing, substituting, replacing, or eliminating high impact carbohydrates to diminish glucose excursions; for example, replacing breakfast oatmeal with unsweetened Greek yogurt and fresh fruit or substituting cauliflower rice for white rice. In unit 3, participants spend 14 days learning how to hasten recovery of glucose excursions by changing the type, intensity, duration, and timing of routine physical activity, such as walking the dog after supper instead of sitting in front of the television or computer. In unit 4, participants learn to continue experimenting with new nutrients and activities, sustain support of significant others, and prevent, anticipate, and recover from relapses resulting from fatigue, life stress, or change in routine. GEM was executed in the context of self-monitoring with diary entries, personal feedback from the CGM and the activity monitor, and automated daily motivational text messages. Following unit 1, there were 5 diary pages, and following units 2-4, there were 14 days of diaries where participants recorded their food and activity choices and how these impacted their glucose excursions.

Figure 1 displays 2 days of pre- and post-CGM data for 1 participant, illustrating how GEM reduces glucose excursions.

After this 6-week GEM program, for the next 3 months, participants continued exploring the principles of GEM with the assistance of their CGM and Fitbit feedback. A 3-month posttreatment assessment involved downloading their CGM data, and repeating the web-based questionnaires. Following completion of these questionnaires, participants rated how “helpful” different elements of the program were on a Likert scale (0=not helpful at all, 1=slightly helpful, 2=somewhat helpful, 3=very helpful, and 4=extremely helpful).

**Figure 2:**
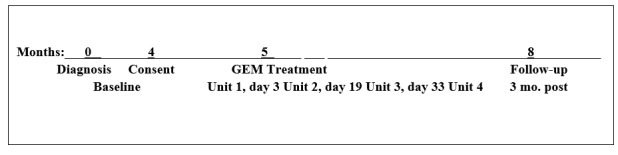
Timeline from diagnoses through follow-up assessment. GEM: glycemic excursion minimization.

Investigators then extracted participants’ diagnostic and post-GEM HbA_1c_ levels, BMI, and prescribed diabetes medications from their medical records (Figure 2).

### Statistical Analysis

SPSS (version 27, IBM Corp) was used to perform 2-tailed paired *t* tests to test for differences in means between GEM participants’ diagnostic (baseline, preintervention) and 3-month postintervention data among the following outcome variables: HbA_1c_ levels, metformin dose, number of CGM readings that were >140 mg/dL, CGM glucose variability expressed as SD values [26,27], BMI, and weight. Pre- and postintervention mean scores were also compared for the following questionnaires or scales: high-carbohydrate food intake, low-carbohydrate food intake, diabetes knowledge, diabetes empowerment, diabetes distress (emotional subscale), diabetes distress (regimen subscale), and depressive symptoms. The Benjamini-Hochberg procedure [28] was used to control for multiple comparisons, correcting for all *P* values in Table 1. Exploratory Pearson correlations were performed between HbA_1c_ levels and both baseline and postintervention variables. There were no missing data, and none of the data sets violated the assumptions of a normal distribution.

## Results

Participants’ mean age at diagnosis was 52 (SD 11.6) years, the mean time between diagnosis and consent was 3.9 (SD 2.5) months, the mean duration of diabetes at postintervention assessment was 8.5 (SD 3.3) months, 40% of participants were female, and 33.3% of participants were non-White. Table 1 provides more detailed baseline data. There was 1 adverse event (contact dermatitis from CGM sensor adhesive) and 2 dropouts (owing to oral surgery and family crisis).

Table 1 illustrates that at 3 months follow-up, GEM led to a significant reduction in HbA_1c_ levels (*P*<.001), with 66.7% of participants qualifying as having their diabetes in remission (HbA_1c_ level <6.5%) [29]. Further, 80% of the participants were classified as Responders (decrease in HbA_1c_ levels of at least 0.5%), with a mean pre-post change in HbA_1c_ levels of –2.3% (SD 1.3%).

**Table 1:** Variables, pretreatment, and 3 months post–glycemic excursion minimization intervention.

Variable				Pretreatment		3 months post–glycemic excursion minimization		*P* value		Baseline correlation with change in hemoglobin A_1c_ levels		Change in correlation with change in hemoglobin A_1c_ levels	
**Primary outcome variables, mean (SD)**													
	Hemoglobin A_1c_ levels (%)			8.0 (1.6)		6.2 (1.1)		<.001^a^			–0.755^b^		
	Metformin (mg/day)			0 (0)		133 (516)		.33					–0.219
**Mechanism variables**													
	**Continuous glucose monitoring data, unblinded weeks 1 and 18, mean (SD)**												
		Percentage of time when continuous glucose monitoring values were >140 mg/dL	23.9 (28.9)		14.5 (22)		.03			–0.012		–0.086	
		Glucose variability	22.4 (10.1)		20.2 (8.3)		.08			0.132		–0.208	
	High-carbohydrate foods			39.6 (21.9)		10.3 (6.8)		<.001^a^			–0.243		0.238
	Low-carbohydrate foods			50.2 (20.8)		48.6 (20.0)		.69			–0.413		0.635^c^
**Secondary benefits, mean (SD)**													
	Diabetes knowledge			15.5 (3.0)		15.9 (3.0)		.53			0.061		0.555^c^
	Diabetes empowerment			31.0 (5.9)		34.6 (3.8)		.06			–0.253		0.251
	Diabetes distress, emotional			2.2 (0.8)		1.8 (1.0)		.10			–0.580^c^		0.015
	Diabetes distress, regimen			2.8 (1.4)		1.8 (0.9)		.03			0.363		–0.052
	Depressive symptoms			6.1 (4.5)		2.3 (3.9)		.001^a^			0.400		0.132
	BMI			36.5 (8.1)		34.4 (8.2)		.002^a^			0.013		0.333

^a^Significant with the Benjamini-Hochberg procedure.

^b^Correlation with *P*<.01.

^c^Correlation with *P*<.05.

Three months post GEM, participants presented reduced CGM readings >140 mg/dL (*P*=.03) and consumed high-carbohydrate foods routinely (*P*<.001). Secondary benefits included reduction of depressive symptoms (*P*=.001) and BMI (*P*=.002).

Table 1 additionally presents correlations of baseline variables with post-GEM reduction in HbA_1c_ levels. Change in HbA_1c_ levels was negatively correlated with baseline HbA_1c_ levels (*r*=–0.755) and emotional diabetes distress (*r*=–0.580). The last column in Table 1 shows how improvement in variables correlated with improvement in HbA_1c_ levels. Greater increase in diabetes knowledge (*r*=0.555) and greater increase in routine intake of low glycemic foods (*r*=0.635) were associated with greater improvement in HbA_1c_ levels.

Reduction in HbA_1c_ levels was only associated with higher baseline HbA_1c_ levels (*r*=–0.755) and emotional diabetes distress (*r*=–0.580). Greater reduction in HbA_1c_ levels was associated with greater pre-post reduction in low-glycemic-load carbohydrate ingestion (*r*=0.635) and improved diabetes knowledge (*r*=0.555).

Posttreatment mean ratings (0-4) for how helpful each of the different elements were: Libre 2 CGM=3.9, Fitbit=3.4, GEM Pocket Guide=2.9, diaries=2.6, GEM Supplement=2.5, and text messages=2.4.

## Discussion

### Principal Findings

Our primary hypotheses were confirmed. Mean HbA_1c_ levels were reduced by 1.8% among all participants, with 67% being classified as having diabetes remission and 80% being classified as responders with a mean HbA_1c_ level reduction of –2.3% among responders. This was achieved with only one participant needing to start taking medication. This participant’s HbA_1c_ level decreased 3.6%, from 12.7% to 9.1%, which was a clinically important improvement that subsequently required the additional introduction of metformin.

Regarding secondary hypotheses, there was a posttreatment decrease in diabetes distress, depression symptoms, and BMI, and a trend toward increased diabetes empowerment.

The strengths of this study are that it was a multicenter, brief, self-administered intervention, which recruited a diverse sample by diverse investigators. The mean change in HbA_1c_ levels of –1.8% by these GEM participants incorporating CGM compares favorably to the –1.0% change in HbA_1c_ levels by a similar group in a randomized controlled trial delivered in a face-to-face format with adults having T2D for an average of 5.3 years of taking diabetes medication [12].

### Limitations

Limitations of our study include a small sample size, no control group, and a limited follow-up duration. Additionally, pre-post change in physical activity, a presumed primary mechanism of GEM, was not monitored in this study. Despite these limitations and multiple positive findings, this is still a pilot study in need of replication with a larger and more diverse sample, which could tease out whether CGM alone would lead to such benefits.

### Comparison With Prior Work

This compares favorably to a mean reduction in HbA_1c_ levels of 1.5% and no psychological benefits with maximum dosage of metformin [30].

Despite being a self-directed program, these results were better than any of our previous efforts [12,15]. This could have resulted from our subject sample consisting of recently diagnosed adults who had not yet begun diabetes medication. This speculation is supported by the UK Prospective Diabetes Study [31], which initiated treatment for newly diagnosed T2D with 3 months of “dietary counseling” alone, with no medication, which led to a reduction in HbA_1c_ levels by ~1%. It could also be because of all participants in this study wanting this intervention—no random assignment. However, it may also be due to the multidimensional nature of the intervention: feedback from CGM and activity monitors, a structured and brief pocket guide and diary, and daily text messages, all of which were considered helpful by participants. It could have been that a reduction in HbA_1c_ levels was highly associated with baseline HbA_1c_ levels, since those with a higher HbA_1c_ level had the possibility of lowering it further, or that greater diabetes distress was associated with greater reduction, as this could reflect greater motivation. Likewise, the greater knowledge acquired about the impact of diet and activity on diabetes, and the greater reduction in routine carbohydrate ingestion were associated with more reduction in HbA_1c_ levels, as these were the hypothesized mechanisms. Reduction in BMI was a secondary benefit and was not correlated with improvement in HbA_1c_ levels.

### Conclusions

GEM augmented with CGM feedback may be an effective initial intervention for adults newly diagnosed with T2D. A self-administered version of GEM may provide primary care physicians and their patients with a new tool to help people recently diagnosed with T2D to achieve remission independent of medication and without weight loss as the primary focus. Future research is needed with a larger and more diverse sample.

## Abbreviations

BG: blood glucose
CGM: continuous glucose monitoring
DSMES: diabetes self-management education and support
GEM: glycemic excursion minimization
HbA_1c_: hemoglobin A_1c_
T2D: type 2 diabetes
